# Navigating optimal treaty-shopping routes using a multiplex network model

**DOI:** 10.1371/journal.pone.0256764

**Published:** 2021-08-27

**Authors:** Sung Jae Park, Kyu-Min Lee, Jae-Suk Yang

**Affiliations:** 1 Graduate School of Future Strategy, Korea Advanced Institute of Science and Technology, Daejeon, Republic of Korea; 2 Johns Hopkins Carey Business School, Baltimore, MD, United States of America; 3 College of Business, Korea Advanced Institute of Science and Technology, Seoul, Republic of Korea; University of Aveiro, NEW ZEALAND

## Abstract

The international tax treaty system is a highly integrated and complex network. In this system, many multinational enterprises (MNEs) explore ways of reducing taxes by choosing optimal detour routes. Treaty abuse by these MNEs causes significant loss of tax revenues for many countries, but there is no systematic way of regulating their actions. However, it may be helpful to find a way of detecting the optimal routes by which MNEs avoid taxes and observe the effects of this behavior. In this paper, we investigate the international tax treaty network system of foreign investment channels based on real data and introduce a novel measure of tax-routing centrality and other centralities via network analysis. Our analysis of tax routing in a multiplex network reveals not only various tax-minimizing routes and their rates, but also new paths which cannot be found by navigating a single network layer. In addition, we identify strongly connected components of the multiplex tax treaty system with minimal tax shopping routes; more than 80 countries are included in this system. This means that there are far more pathways to be observed than can be detected on any given individual single layer. We provide a unified framework for analyzing the international tax treaty system and observing the effects of tax avoidance by MNEs.

## Introduction

One of the major points on the agenda of the Organization for Economic Cooperation and Development (OECD) in past decades has been tax avoidance by multinational enterprises (MNEs). Countries lose about 100–240 billion US dollars in tax revenues annually due to global profit shifting, equivalent to between 4% and 10% of global corporate income tax revenues [1–4]. MNEs exploit provisions of interjurisdictional tax arrangements, not hesitating to direct income flows surreptitiously or through various financial vehicles [5–8]. Tax avoidance by big companies such as Google, Apple, Facebook, Microsoft, and Starbucks has been reported in the media over the past few years, but no fundamental solution has yet been found [9].

The international tax treaty system is a complex regime composed of thousands of bilateral tax treaties. Treaty shopping, in this context, refers to the conduct of MNEs that deliberately seek to benefit from the statutes of a tax treaty by making foreign investments [5, 6, 10]. Though there are several anti-treaty shopping policies such as beneficial ownership, main purpose test, and limitation on benefits provisions, the current tax system prevents neither the establishment of paper companies in countries or regions where favorable tax rates are imposed under tax treaties, nor the avoidance of taxes using differences between tax laws and treaties [6, 11–14]. As a countermeasure, the OECD 2015 final report to prevent Base Erosion and Profit Shifting (BEPS) was adopted at the G20 Summit held in Turkey on November 16, 2015 [15]. The BEPS report focused on preventing abuse of tax treaties, neutralizing the effects of hybrid mismatch arrangements, limiting base erosion involving interest deductions, preventing harmful tax competition, and documenting transfer pricing. However, the efficacy of these measures is still questioned.

A tax treaty is basically a bilateral treaty between two countries. Treaty abuse refers to the case where an individual or corporation that is not eligible for tax reduction benefits under the tax treaty establishes intermediary entities that are eligible, thus enabling them to receive tax reductions or exemptions unfairly. MNEs avoid taxes by implementing the following business strategies: transferring income from the global market to countries with lower tax rates in the form of dividends, interest, and royalties, or by using the interest deduction system. It was reported that around 40% of MNE profits worldwide were artificially shifted to tax havens in 2015 [16]. MNEs also take advantage of loopholes between domestic tax laws and tax treaties between countries in their cross-border transactions. Capitalizing on discrepancies, MNEs can create transactional structures that allow them not to pay taxes in any country in the world.

Treaty shopping involves searching for favorable tax treaties for the purpose of tax avoidance. This is possible because there are significant differences between tax treaty rates and domestic withholding tax rates. Hybrid mismatch arrangements can be made that involve changing the form of income (dividends, interest, and royalties) as a tool for tax avoidance. In addition, the complexity of tax routing makes it difficult for governments to track and respond to treaty-shopping behavior. Huge profit shifting by MNEs connotes this problem.

### Previous literature on tax avoidance

As a complex network, the international tax treaty system should be considered using a complex network approach. However, only a few pioneering studies have examined the tax system and avoidance behavior using network analysis [17–19]. Hong constructed a tax rate matrix of dividends to represent a network of tax treaties between 70 countries, creating a computational algorithm to study tax-minimizing investment routes in the network [17]. He found a treaty-shopping rate, defined as the difference between the direct route and a tax-minimizing route, of about 3.66%p on average [17]. van’t Riet and Lejour found that treaty shopping leads to a reduction of the tax burden on repatriated dividends of about 6%p. In that study, the UK, Luxembourg, and the Netherlands were the most important conduit countries in the tax network [18]. They also found that unlike tax haven financial centers, low-tax havens did not have an important role in reducing dividend repatriation taxes [18]. Petkova et al. investigated the effects of tax treaties on foreign direct investment (FDI) after controlling for their relevance in the presence of treaty shopping and showed that relevant tax treaties increased FDI by about 18%p [19]. These previous studies, however, all had some limitations, as follows:

1. The analyses only included a limited number of tax treaties. Optimal treaty shopping routes cannot be captured with limited data.
2. They focused on dividends; interest and royalties were not included in the analyses.
3. The authors assumed that the intermediate station in a given network route includes only one [18] or two [17, 19] countries. One recent study [20] expanded the scope of its analysis of investment (ownership) structures to three countries. However, in reality, an intermediate station in a tax treaty network can include any number of countries.

Treaty shopping as discussed in the OECD/G20 BEPS report is closely related to large MNEs. The global transaction structures of companies such as Apple, Google, and Starbucks [21, 22] and international investment channels such as Lone Star [23] are multi-tiered and complicated. These companies are statistical outliers; thus, though the number of companies engaging in treaty shopping is not many, the scale of tax avoidance is very large. Moreover, real tax avoidance occurs through use of multiple income channels (dividends, interest, and royalties) rather than single channels in isolated incidents [24]. In many cases, companies choose a kind of portfolio approach or strategy of mixing multiple types of income. In the OECD Action 2 Final Report, for example, hybrid mismatch cases included hybrid instruments (for example, treating an instrument as debt in one territory and equity in another) and hybrid transfer arrangements (mismatches resulting from the differing ways that parties treat arrangements such as stock loans and repos for tax purposes). Therefore, to investigate the complexity of the system fully, a multiplex network framework is required. Multiplex network studies have been conducted of the transport system, as follows: navigating the shortest paths in a multiplex network to model real transportation systems [25], controlling congestion [26], observing navigability on random failures [27], and validating origin-destination demand from empirical data [28]. The tax treaty system is an ideal setting in which to apply a multiplex network approach.

### Multiplex network of the tax treaty system

Based on the background provided here and to address the limitations of previous works, in this study, we investigate the issue of treaty shopping with a comprehensive list of countries as well as multiple channels of taxable income. Since MNEs use multiple channels to navigate optimal treaty shopping routes, we believe that multiple channels of the tax treaty system can best be modeled using a multiplex network framework [29–32]. In such complex systems, several dynamic phenomena may be observed, such as catastrophic cascading failures [33, 34], super-spreading events in epidemic processes [35, 36], and synergistic activations in behavior spreading [37–39], which cannot be reproduced in a single-layer network framework.

**Fig 1:**
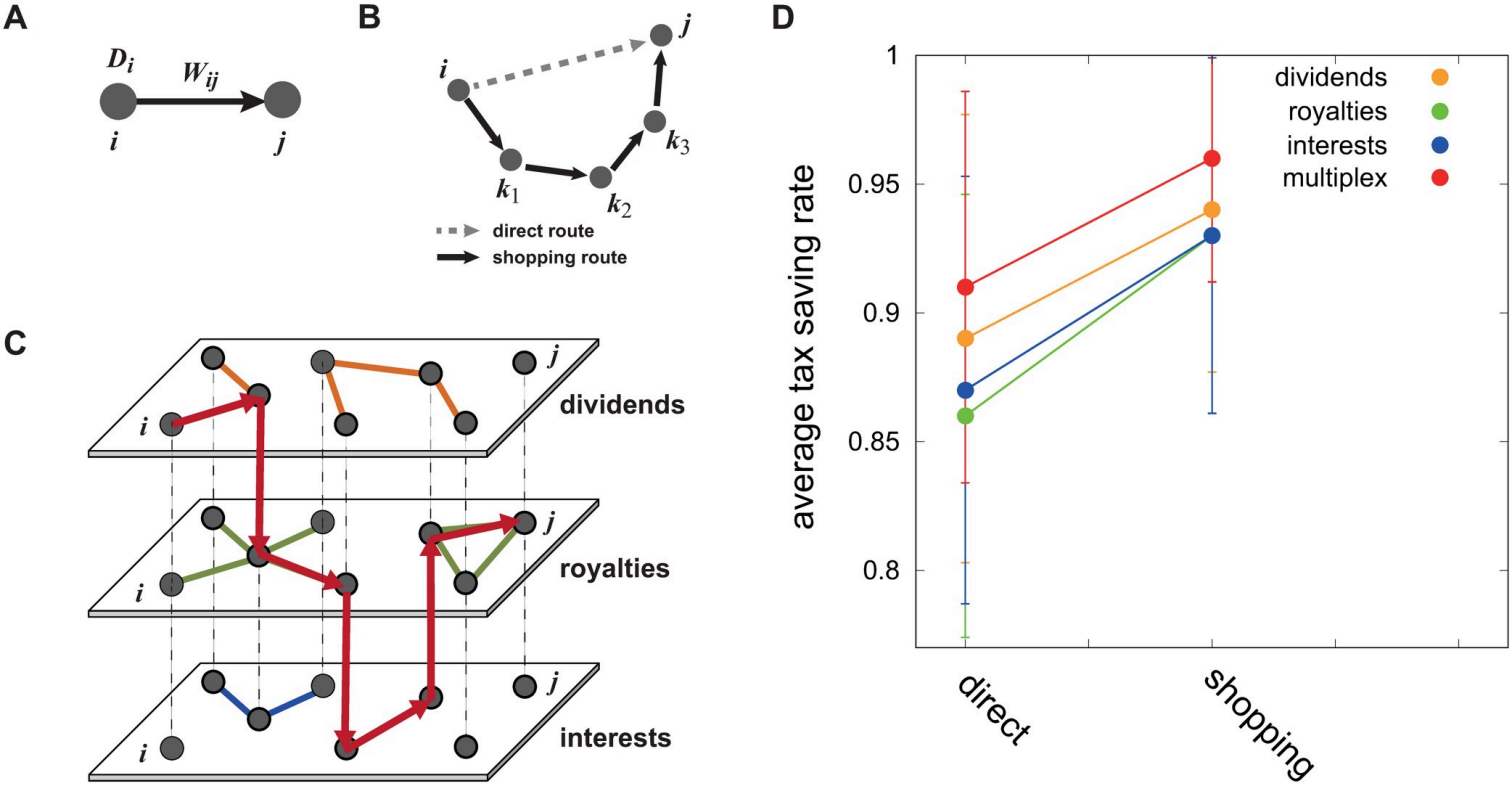
Treaty-shopping routes and the multiplex network framework. (A) The components in a dyadic tax treaty between two countries *i* and *j*. *D*_*i*_ denotes the domestic tax rate of a country *i* and *W*_*ij*_ means the withholding tax rates between the two countries. (B) Schematic example of tax-routing routes: direct (dotted arrow) and treaty shopping routes (solid arrows). (C) Schematic diagram of multiplex tax treaty networks. Layers 1, 2, and 3 represent different types of income (dividends, royalties, and interest, respectively). In identifying tax-minimizing routes from country *i* to country *j*, we see there are no routes between these two countries when the system is considered as as a series of isolated single layers. However, when we consider it as a multiplex network system, we can find the optimal route by crossing layers (depicted in red). Additional tax-minimizing routes may also be available in this multiplex framework of the tax treaty system. (D) Tax-routing centrality value of single layers (dividends, royalties, and interest) and the multiplex network for both route types (direct and shopping).

To construct a multiplex network of the tax treaty system, we collect and analyze data on tax treaty rates and domestic tax rates for 217 countries in the 2015–2016 fiscal year (see S1 Appendix for details; note that no interest deduction system is included in the model). When a company tries to move its investment resources from country *i* to country *j*, there are two main considerations: the domestic tax rate of country *i* (*D*_*i*_) and the bilateral tax treaty rate between the two countries *i* and *j* (*W*_*ij*_) (Fig 1A). Following international taxation law, the tax rate applied to the company is the lower of the two given rates *D*_*i*_ and *W*_*ij*_. Additionally, corporate income tax and company maintenance fees are associated with each event, so we consider one more cost *α* as a simple proxy for the additional tax rate of individual companies. Finally, we define the direct route tax rates (DTR) between countries *i* and *j* as

$$\begin{array}{c} DTR_{ij}=\text{Min}\left(D_{i},W_{ij}\right)+\alpha . \end{array}$$ (1)

where the value of *α* is given by 0.01 (as 1%) for a certain minimum value.

Moreover, in the tax treaty system, there are countries with zero-level domestic tax rates (*D*_*i*_ = 0), such that the value of DTR is always minimal (*DTR*_*ij*_ = *α*) to all target countries *j* when a company departs from a given source country *i*. We identify those countries as “minimum tax-rate countries”; notably, these countries play a crucial role in navigating optimal treaty-shopping routes because they can reach any other countries at minimal cost *α*. In reality, some (but not all) of them are traditional “tax-haven” countries designated by multiple organizations or research firms (see S1 Appendix for more details).

Due to the highly complex nature of the tax treaty system and differences in rates among countries, direct routing from country *i* to country *j* (Fig 1B, dotted arrow) is not always optimal. When a company in country *i* decides to select a route to country *j*, even if the chosen path requires more than one step (Fig 1B, solid arrows), it may be the most efficient route, the most rational choice in terms of avoiding taxes. In this study, we identify optimal routes by adjusting the algorithm to find the shortest paths between nodes in networks.

Moreover, since we consider three income layers (dividends, interest, and royalties) of the tax treaty system, the optimal path may include different combinations of all three. Therefore, the problem can be stated as finding the shortest paths in a multiplex network when the optimal route may necessitate crossing different network layers. To construct a multiplex network of the tax treaty system, we therefore consider each layer as complementary to the navigating issue, such that two nodes that are not connected in a single layer may be linked via other layers (Fig 1C). The possibility that optimal paths may exist across different income layers implies that finding the optimal treaty shopping route is more complex than can be modelled by considering only a single income layer.

## Materials and methods

### Data sources

The tax data used in this paper is a compilation of data on tax treaty rates and domestic tax rates in each country in reports published by PwC, Deloitte, KPMG, and EY in the 2015–2016 fiscal year. Details of the sources of the collected data are presented in the S1 Appendix, where the dataset we used is also provided.

### After-withholding-tax-income rate (ATR)

By determining the rate of savings due to tax routing in each network, the effects of treaty shopping can be quantified. When moving funds from a specific country *i* to country *j*, a company chooses a direct route; therefore, the after-withholding-tax-income rate (*ATR*) is

$$\begin{array}{c} ATR_{ij}^{direct}=1-DTR_{ij} \end{array}$$ (2)

that is, when a company moves a fund with initial value *F*_*initial*_ from country *i* to country *j* directly (Fig 1B, dashed arrow), then the final value of the fund after withholding tax becomes $F_{after}=F_{initial}\times ATR_{ij}^{direct}=F_{initial}\left(1-DTR_{ij}\right)$, where *DTR*_*ij*_ denotes the direct tax rate between *i* and *j* (Eq 1).

Now, let’s assume that a company tries to find a route to maximize tax savings through treaty shopping (Fig 1B, solid arrows). Given the tax minimizing route (*i* → *k*_1_ → *k*_2_ → *k*_3_ → *j*), then the *ATR* becomes

$$\begin{array}{c} ATR_{ij}^{shopping}=\left(1-DTR_{ik_{1}}\right)\left(1-DTR_{k_{1}k_{2}}\right)\left(1-DTR_{k_{2}k_{3}}\right)\left(1-DTR_{k_{3}j}\right) \end{array}$$ (3)

where *n* is the number of places (countries) through which the fund arrives at destination *j*. As above, the initial fund *F*_*initial*_ becomes $F_{after}=F_{initial}\times ATR_{ij}^{shopping}=F_{initial}\left(1-DTR_{ik_{1}}\right)\left(1-DTR_{k_{1}k_{2}}\right)\left(1-DTR_{k_{2}k_{3}}\right)\left(1-DTR_{k_{3}j}\right)$

In most cases, companies search for routes that enable them to avoid paying taxes; that is, $ATR_{ij}^{direct}<ATR_{ij}^{shopping}$. Generally, companies choose a treaty-shopping route (multiple steps with *ATR*^*shopping*^) rather than moving their funds directly. Thus, we can say that MNEs seeking tax benefits through treaty shopping find the route that maximizes the value of $ATR_{ij}^{shopping}$ and move their funds accordingly.

### Algorithm for finding tax-minimizing routes

To identify tax-minimizing routes from country *i* to country *j* on each single network layer, we apply the Dijkstra’s algorithm that finds the shortest paths between two nodes in a given network [40]. Since the *ATR* is determined by the multiplicative method of calculating tax rates (Eq 3), we perform the logarithmic transformation to all link weights 1 − *DTR*_*ik*_ in the additive way, as follows:

$$\begin{array}{c} DTR_{ik}^{\prime }=\text{log}{\left(1-DTR_{ik}\right)}^{-1} \end{array}$$ (4)

then, we must find the minimum value of $ATR_{ij}^{\prime }$ (*i* → *k*_1_ → *k*_2_ → ⋯ → *k*_*n*_ → *j*),

$$\begin{array}{c} ATR_{ij}^{\prime shopping}=DTR_{ik1}^{\prime }+DTR_{k_{1}k_{2}}^{\prime }+\cdots +DTR_{k_{n}j}^{\prime } \end{array}$$ (5)

Then, we apply the standard Dijkstra’s algorithm to find the shortest paths between two countries. After identifying the transformed $ATR_{ij}^{\prime shopping}$, we retransform the value as $e^{-ATR_{ij}^{\prime }}$, after which we can obtain the $ATR_{ij}^{shopping}$ with the original Eq 3.

For the multiplex network case, we overlay all network layers into one composite layer and then apply the same algorithm as in the single-layer case [25, 26]. When there are overlapped links between two countries across a single layer, we choose the minimum weights among them, as follows:

$$\begin{array}{c} DTR_{ij}^{multiplex}=\text{Min}\left(DTR_{ij}^{dividends},DTR_{ij}^{royalties},DTR_{ij}^{interest}\right). \end{array}$$ (6)

### Tax-routing centrality (TRC)

Since the value of $ATR_{ij}^{shopping}$ can be obtained using all possible pairs of dyadic relations, we can identify and compare the shopping effects (and tax-savings effects as differences between direct and shopping routes) of individual countries by averaging the overall routes starting from a given country, as follows:

$$\begin{array}{c} TRC_{i}^{direct}=\frac{1}{N-1}\sum_{j\neq i}ATR_{ij}^{direct}, \end{array}$$ (7)

$$\begin{array}{c} TRC_{i}^{shopping}=\frac{1}{N-1}\sum_{j\neq i}ATR_{ij}^{shopping}, \end{array}$$ (8)

depending on whether a company chooses the direct routing strategy (*TRC*^*direct*^) or decides to go treaty shopping (*TRC*^*shopping*^). We label this variable tax-routing centrality (*TRC*), since the higher the value, the higher the savings when funds are moved from a given source country. We can consider this a measure of centrality, as it is similar to the closeness centrality of weighted networks. Also, we can quantify the shopping effect (and tax-savings effect) of the network as a whole by determining the sum of all TRC measures for all countries, as follows:

$$\begin{array}{c} TRC^{direct}=\frac{1}{N}\sum_{i}TRC_{i}^{direct}, \end{array}$$ (9)

$$\begin{array}{c} TRC^{shopping}=\frac{1}{N}\sum_{i}TRC_{i}^{shopping}, \end{array}$$ (10)

We can then compare the tax-savings effects of different income layers in the network, that is, dividends, interest, and royalties, with a multiplex network including all three layers.

### Betweenness centrality

Betweenness centrality is a measure in network analysis that quantifies how many shortest paths in a network pass through a given node. Therefore, not only is tax-routing centrality useful as defined above, but betweenness centrality is also useful to determine the importance of each node (country) in the problem of navigating treaty shopping.

Betweenness centrality is defined by

$$\begin{array}{c} B_{i}=\sum_{s\neq i\neq t}\frac{\sigma_{st}\left(i\right)}{\sigma_{st}} \end{array}$$ (11)

where *σ*_*st*_ is the total number of shortest paths from node *s* to node *t* and *σ*_*st*_(*i*) is the number of those paths that passes through node *i*. In the problem of navigating treaty shopping, we consider the measure of *σ*_*st*_ as the total number of savings routes from node *s* to node *t*, and *σ*_*st*_(*i*) is the number of those routes that passes through node *i*.

### Minimum treaty-shopping routes and the strongly connected component (SCC)

In a directed network, a strongly connected component (SCC) can be identified when there exists a path in both directions between each pair of nodes in a network. We define a giant strongly connected component (g-SCC) as one that contains the maximum number of nodes among all components.

In the tax treaty network, there exists a dyadic relation with the minimum rate, that is, *DTR*_*ij*_ = *α*, between country *i* and country *j*. Interestingly, the existence of this minimum-rate relationship implies that we can identify the shopping routes between country *i* and country *j*, such that $ATR_{ij}^{shopping}=\alpha$. This means that companies would save almost 100% (99% in our model) of their funds from being taxed when moving them. Similarly, we can also define the g-SCC in the tax treaty system using minimum tax-rate routes. In doing so, we can observe any relations between countries through which companies can move their funds freely with minimal loss of funds due to taxation.

## Results

From the measures introduced in the Materials and methods section, we first observe the treaty-shopping effect in tax treaty networks. For a given pair of nodes (countries), we can measure the *ATR* for both direct and shopping cases, summarizing the overall effect in Eqs 9 and 10 respectively (Fig 1D). In the case of direct routes, all *TRC* values are close to 0.87 (exact values are presented in the S1 Appendix), whereas the values are greater than 0.9 in shopping route cases (Fig 1D). For the example of dividends, the shopping route rate is 0.94, but it is 0.89 in the direct case, indicating that the shopping route would provide a savings of almost 5%p in taxes using this strategy. For example, a company trying to move 500 million dollars of dividends via treaty shopping can save almost 25 million dollars in this examples. For the other income layers, the savings effect is similar (7%p in royalties, 6%p in interest; see S1 Appendix for more detail).

Moreover, in the multiplex network, the savings effect is even more amplified. The average *TRC* exceeds 0.96, indicating that a company can save an additional 2–3% in taxes compared with the case of single income layers. This is not a small amount of money for a company. Therefore, we can assume that companies will search for optimal shopping routes from the multiplex network perspective (that is, combining or making a portfolio of more than one income strategy) when moving funds internationally. In other words, MNEs have significant motivation to engage in treaty shopping.

**Fig 2:**
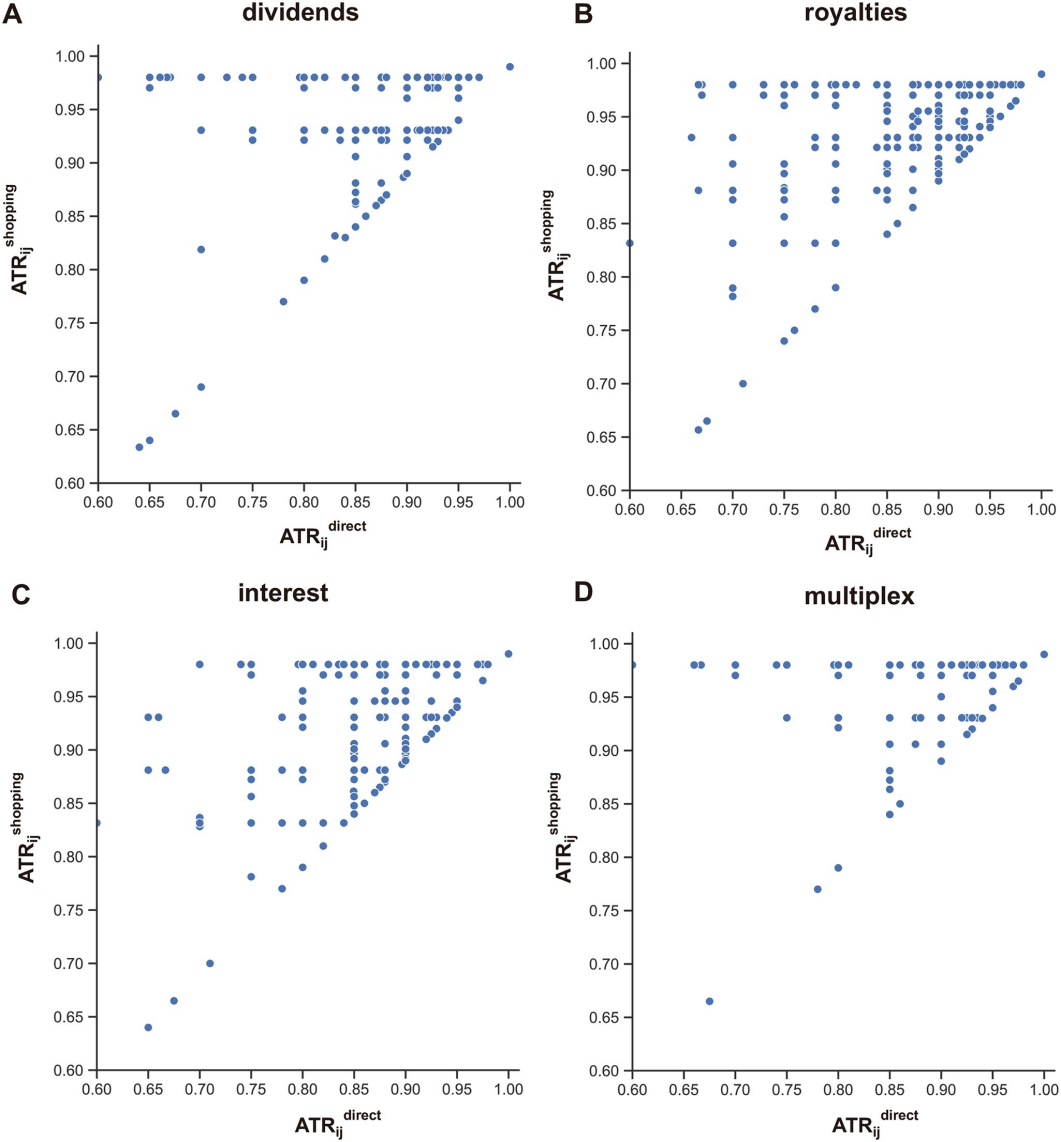
Comparison between direct and shopping routes for (A) dividends, (B) royalties, (C) interest, and (D) the multiplex network. The inset tables present values for the top 10 countries with the largest differences between direct and shopping routes in each layer.

From the observations for *TRC* in the overall network, we can quantify and compare the savings effects of treaty shopping for each income layer and the multiplex network. We then conclude that the multiplex network provides additional savings opportunities for MNEs to avoid paying taxes. In addition, we can measure the *TRC* for individual countries and evaluate their roles in global treaty-shopping events. In Fig 2, we compare the *TRC* measures of individual countries for direct and shopping routes in each income layer. Most countries are above the diagonal line in the figure (*y* = *x*), which means that the *TRC* value increases when a company chooses a treaty shopping route starting from that country. Moreover, a large number of countries (more than 50%) have a *TRC*^*shopping*^ value of 0.99, indicating that they would provide minimum tax-rate tax routing (that is, the companies moving funds pay 1% of the tax-rate) in treaty-shopping cases. In multiplex networks, this savings effect is magnified, as we find that in the network as a whole (Fig 1D), the number of countries with a *TRC*^*shopping*^ value of 0.99 has significantly increased (164 countries, more than 75% of the total number of nations). By comparing the *TRC* value for each country for direct and shopping routes, we can quantify the benefits that can be obtained through choosing tax avoidance routes from that country, and compare countries. The top 10 countries with the largest differences between direct and shopping routes in each layer are presented in the tables in Fig 2.

**Fig 3:**
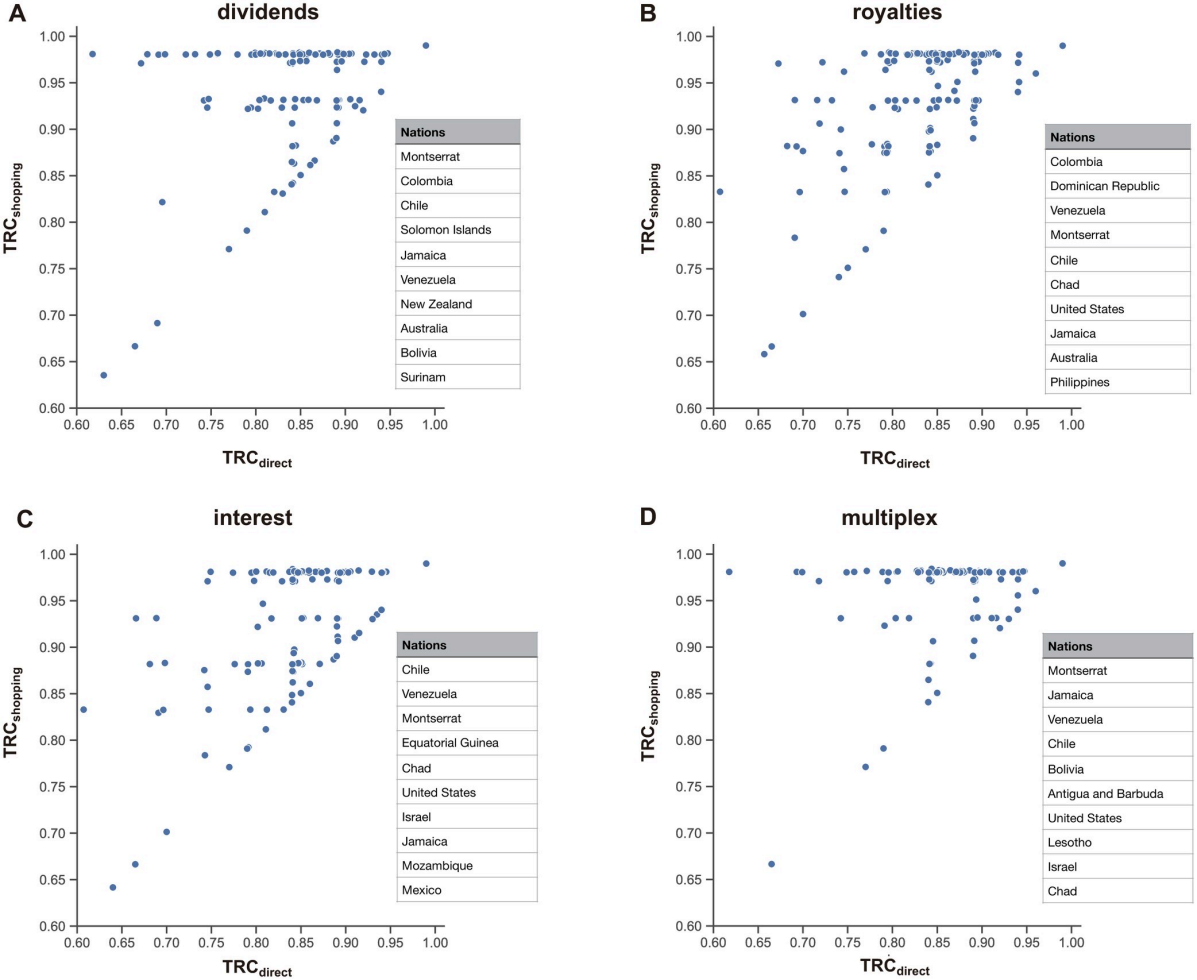
Pairwise comparison between direct and shopping routes for (A) dividends, (B) royalties, (C) interest, and (D) the multiplex network.

Additionally, we perform a pair-wise comparison between direct ($ATR_{ij}^{direct}$) and shopping routes ($ATR_{ij}^{shopping}$) (Fig 3). The patterns of the scatter-plots are similar to the results for their *TRC* counterparts in Fig 2, although the points are greater.

**Fig 4:**
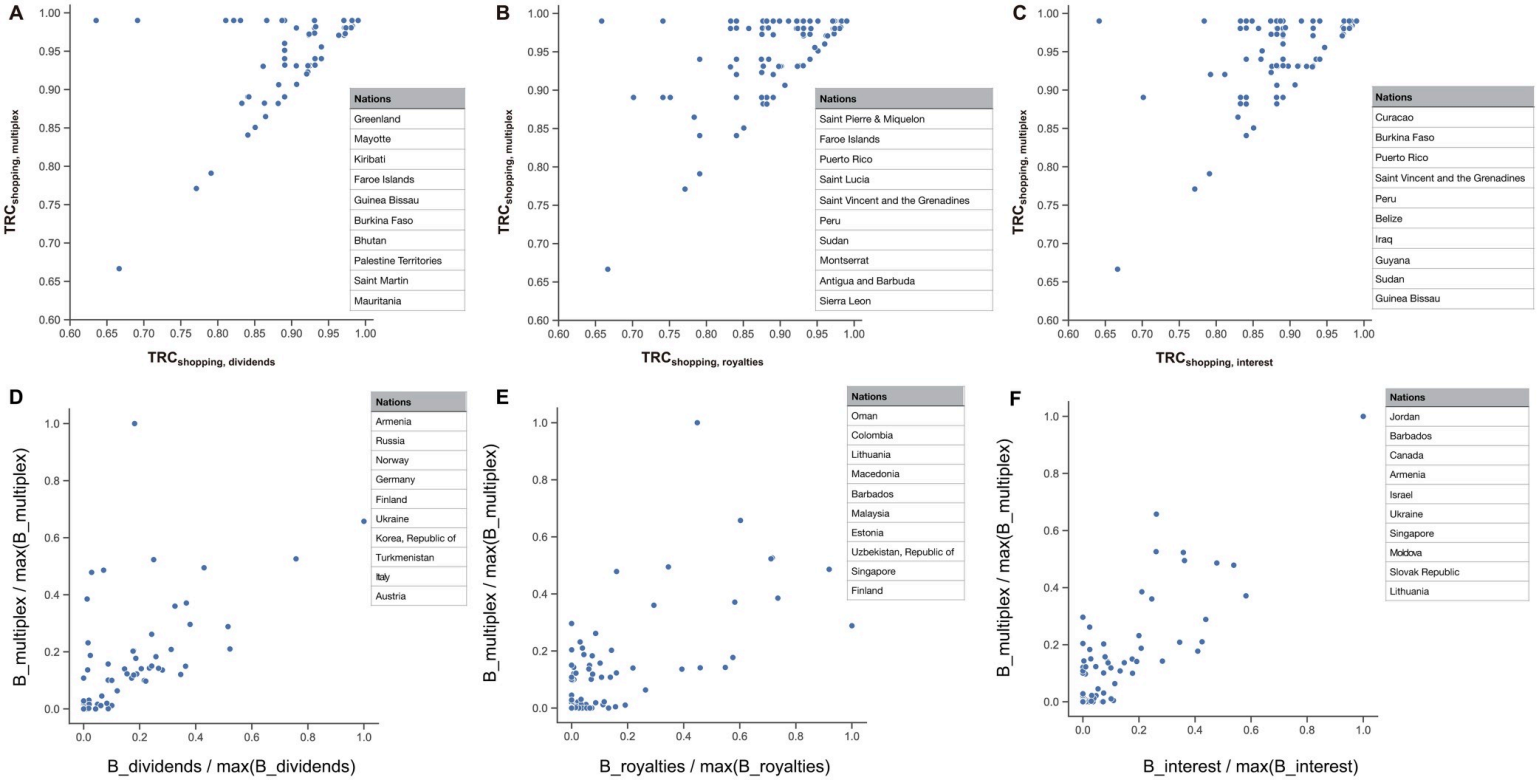
Comparison between the multiplex network and each single layer. Comparison of *TRC* values (A-C) and betweenness centrality (D-F) between the multiplex network and three income layers. The top 10 countries with the highest ratios between the multiplex network and each single layer are presented in the inset tables in order from largest to smallest values.

Now we compare the *TRC* values of the multiplex network with those of each single layer (Fig 4A–4C). First, for all countries, the *TRC* values in the multiplex layer are equal to or higher than those in the single layer. This shows that if a company engages in treaty shopping through the multiplex network, the tax savings are greater. By observing differences between the *TRC* values in the single layers and the multiplex network, it is possible to quantify how much tax savings can be achieved when moving money from country to country through treaty shopping in the multiplex network. The top 10 countries with the greatest savings effects (the ratio between the two measures; *TRC*^*multi*^/*TRC*^*single*^) are listed in the tables in Fig 4. For example, in the case of Greenland in the dividends layer, if a company engages in treaty shopping using only dividends, the average *TRC* value is about 0.63, and a high tax of 37% on average is imposed. However, if the company moves funds using the multiplex network, thereby, optimizing the route among all three income layers, then the *TRC* value is 0.99, and minimal taxes are paid. These routes are not captured when only a single layer is considered, which results in underestimation of Greenland’s role as a treaty-shopping route.

As another measure of centrality, we now compare betweenness centrality for all three single layers and the multiplex network (Fig 4D–4F). In this case, the role of each country as a stopover can be measured by comparing the degree to which it is used as a stopover in each single layer and the multiplex network. In this way, we calculate the differences between their betweenness centrality values. We present values for 10 countries in order from largest to smallest in Fig 4. For example, in the case of Germany in the dividends layer, the route considering only dividends is not widely used as a stopover, but it is widely used as a stopover in the multiplex network. In other words, considering only a single layer could lead to underestimation of the role of Germany in treaty shopping. Most existing studies of activities involved in designating and finding tax havens examined only one single layer. However, clearly treaty-shopping activities cross income types; MNEs make great efforts to find complex routes through which to move funds. In fact, there may be unidentified routes or countries located at the center that have not been identified in previous studies. Using our method with the multiplex network, these activities can be clearly identified in more detail, as seen in Fig 4.

**Fig 5:**
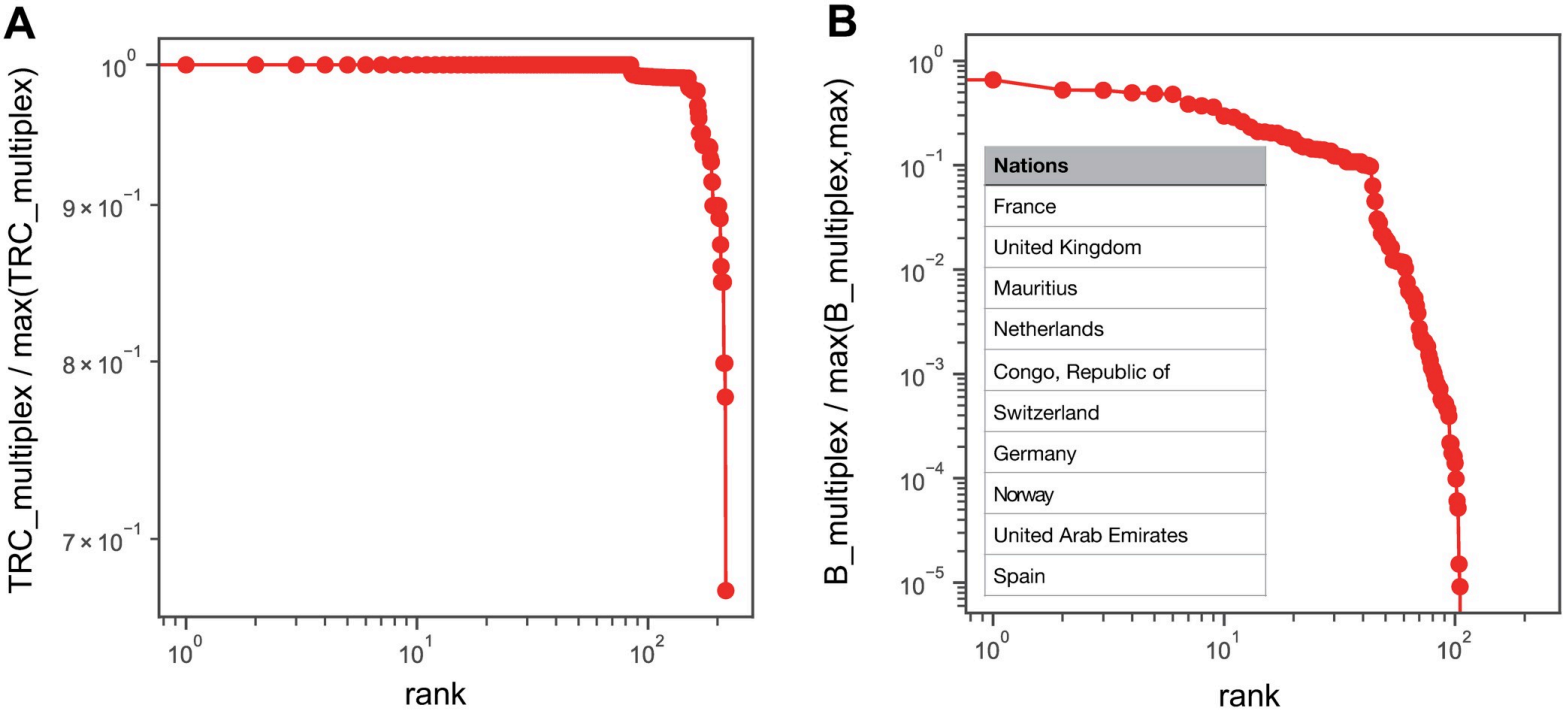
Rank distributions of centralities of the multiplex network. Rank distribution of TRC (A) and betweenness centrality (B) in the multiplex network.

In Fig 5, we consider the rank distribution of the values for *TRC* and betweenness centrality in the multiplex network. The peculiarity of the *TRC* distribution is that a significant number of countries are observed to have a maximum *TRC* value, which means that all of them have a *TRC* value of 0.99 (i.e., these are countries to which companies can move funds without paying taxes). Out of 217 countries in total, this is true for 164 countries. Companies seeking to move funds find that it is possible to move money to any country in the world through a so-called “minimum tax-rate route” that allows them to pay minimal taxes. Compared to the case of using a single type of income to move funds (dividends: 140, royalties: 117, interest: 116), when using the multiplex network, 20—50 more countries are identified as minimum tax-rate routes (Fig 5A). In addition, when comparing the ranking in terms of betweenness centrality, France, the United Kingdom, Mauritius, and the Netherlands are the top places, as these countries are located on preferred stopover routes for treaty shopping (Fig 5B).

**Fig 6:**
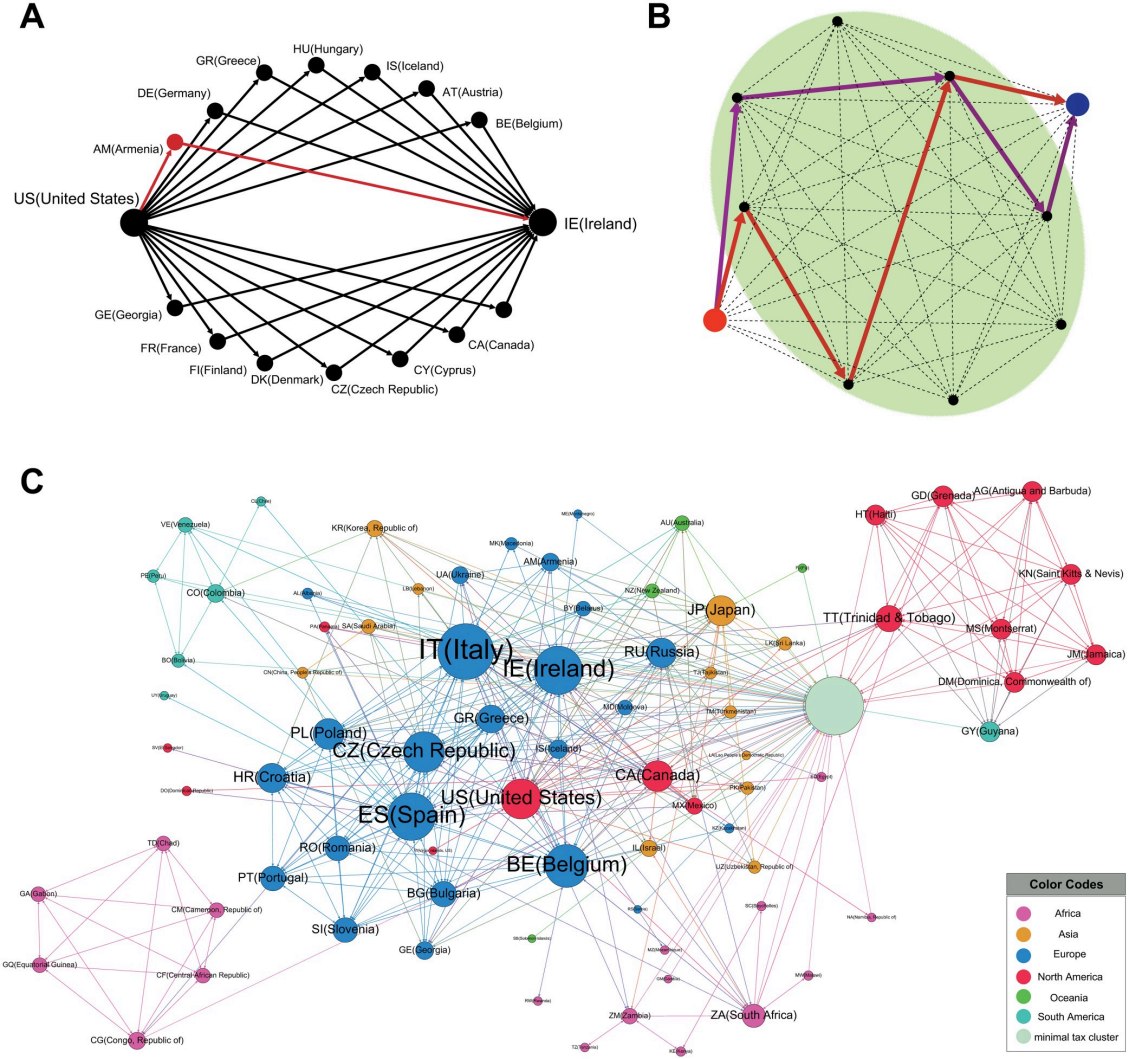
The giant strongly connected component with zero-rate paths. (A) Example of tax-routing routes from the United States (US) to Ireland (IE). The figure presents the routes that can be found in each single layer (black) and those that can only be found in the multiplex network (red). (B) Due to the existence of countries with minimum withholding tax rates, there is a tremendously large number of routes with minimum tax-rates within a cluster (green area) and several steps (indicated by red and purple arrows). (C) The giant strongly connected component (g-SCC) consists of two countries with minimum tax-rates. Colors of nodes represent continents.

While the results so far have demonstrated possible tax-minimizing routes in the multiplex networks for each country from a micro perspective, Fig 6 shows what new insights the multiplex network approach can provide from a more macroscopic perspective. When a multiplex network that includes three layers together among the tax-minimizing route options is considered, not only do we see the results of a simple combination, but we also see new tax-minimizing routes that could not be found previously. For example, companies moving funds from the United States to Ireland may choose tax-minimizing routes that go through a wide variety of countries, as shown in Fig 6A; among them, new routes can be found by utilizing the multiplex network (e.g., the route through Armenia). Therefore, by considering the multiplex network in this way, it is possible to find new tax-minimizing routes that could not be found by utilizing only a single-layer framework. This finding has implications both for companies seeking such novel shopping routes and for governments or international organizations that want to prevent and regulate such treaty shopping.

Furthermore, some countries have minimum domestic withholding tax rates; in these countries, there is a mechanism in place that allows them to send funds at a minimum rate, no matter which country they target. We name the cluster consisting of these countries the “minimum tax-rate cluster”. If a company in a given country not yet in the minimum tax-rate cluster can move funds to a country in the minimum tax-rate cluster, it gains access to a tremendous number of shopping routes in that cluster, as indicated by the red and purple routes in (Fig 6B).

Finally, we also identify bilateral zero-rate tax treaties between all countries (*W*_*ij*_ = 0), not specifically countries that have zero-rate domestic withholding tax rates. In these cases, we extract from the entire network only the links consisting of these zero-rate relationships, observing a strongly connected component in those relationships (Fig 6C). In this strongly connected component, any country belonging to the component can be connected to one or more countries with minimum tax rate treaty-shopping routes. Surprisingly, a large number of countries (80 countries) belong to this component. Because there are so many countries in this component, there is a wider range of routes for minimizing taxes.

## Discussion

We comprehensively analyzed the tax treaty network system in this study. Unlike previous studies [17–19] analyzing tax data from around 100 countries/regions in terms of dividends only, we used tax data from 217 countries/regions for dividends, interest, and royalties. In addition to examining single routes in our network analysis, we also used a multiplex framework (Fig 1C) to examine combinations of income forms (dividends, interest, and royalties). As a result, we found more routes for tax minimization through multiplex detours and more dramatic tax-savings effects than is possible with a single-route network analysis (Figs 1–3).

We measured the treaty-shopping effect in the tax treaty network by comparing direct route with various possible detours. In the multiplex network, a large tax-savings effect was found for royalties (7%p), dividends (5%p), and interest (6%p) (Fig 1D). Using the multiplex network, companies can change the form of income on the network path to obtain an additional 2–3%p in tax savings. This is a very attractive amount for MNEs. According to previous studies [1–4], the amount of tax avoidance due to global profit shifting has reached 100–240 billion US dollars annually over the past 5 years, equivalent to between 4% and 10% of global corporate income tax revenue. Zucman reported that around 40% of the profits of MNEs worldwide were artificially shifted to tax havens in 2015 [16]. Our study identified the specific tax avoidance pathways that back up the findings of previous studies.

In our study, tax-routing centrality, which represents the amount of tax savings achievable via treaty shopping, was measured and the results presented by income type (dividends, interest, royalties, or the multiplex network). In treaty shopping, many countries find routes with a *TRC*^*shopping*^ value of 0.99, thus allowing them to pay minimal taxes; in this study, this value was found for 164 countries in the multiplex network. This means that there are a great many routes that can be taken to avoid paying taxes. The specific role of individual countries in treaty shopping is sometimes not elucidated in analyses of single-route networks. However, we were able to detect these roles in our multiplex network analysis. In the case of Greenland, for example, it was found that the pathway of dividends plays a very important role in tax avoidance, as evidenced by the results of the multiplex network analysis (Fig 4A).

In our study, several interesting points were found regarding the United States, where the scale of tax benefits from treaty shopping was large in terms of royalties, interest, and multiplex incomes; this was not true only for a single channel, dividends (Fig 3B–3D). Additionally, analyzing the investment pathways from the United States to Ireland reveals the pathways through Armenia that would not appear in a single-route network analysis, but was very evident in our multiplex network analysis (Fig 6A).

Our study confirmed that treaty-shopping routes are diverse. First, we created a minimum tax-rate cluster by gathering data for countries with a withholding tax rate of 0% (node cost 1%) through which MNEs can move their funds while paying minimal taxes. Then, we found that various shopping routes could be created using these countries (Fig 6B). These countries are not generally known as tax havens. Next, we constructed a network including only the paths with a 0% tax rate (node cost 1%) for domestic taxes or treaty taxes on dividends, interest, and royalties from 217 countries. In this analysis, we observed a strongly connected component in these relationships (Fig 6C). All countries in this SCC have routes to one or more countries through countries with a 0% tax rate (node cost 1%). It is very interesting that 80 countries belong to this component; it suggests that there are more diversified pathways for tax minimization than the ones identified to date. Our findings signal governments and companies about various treaty-shopping routes that may have been heretofore unknown.

## Supporting information

S1 Appendix Description of the international tax treaty system. Detailed description of the international tax treaty system and observations.
(ZIP)

S1 Fig Comparison between direct and shopping routes for restricted transition between layers. (PDF)

S1 Data Tax treaty data of three income types. (ZIP)

S2 Data Corporate tax rates. (ZIP)

S3 Data Withholding tax rates. (ZIP)
