# Correction: Navigating optimal treaty-shopping routes using a multiplex network model

**DOI:** 10.1371/journal.pone.0269581

**Published:** 2022-06-02

**Authors:** Sung Jae Park, Kyu-Min Lee, Jae-Suk Yang

There is an error in point 3 of the numbered list in the Previous literature on tax avoidance subsection of the Introduction. The correct statement is: The authors assumed that the intermediate station in a given network route includes only one or two countries [17, 19]. One recent study [20] expanded the scope of its analysis of investment (ownership) structures to three countries and van’t Riet and Lejour [18] expanded it to four intermediate countries. However, in reality, an intermediate station in a tax treaty network can include any number of countries.
