# Supplementary material for: Navigating optimal treaty-shopping routes using a multiplex network model
Source: PLoS One. 2021 Aug 27;16(8):e0256764. doi: 10.1371/journal.pone.0256764 (PMC8396775; doi:10.1371/journal.pone.0256764)
Supplement: S1 Appendix — Detailed description of the international tax treaty system and observations. (ZIP) [file pone.0256764.s001.zip › S1_Appendix.pdf]

S1 Appendix Supplementary information

Navigating Optimal Treaty Shopping Routes Using A Multiplex Network Model
by Sung Jae Park, Kyu-Min Lee, and Jae-Suk Yang

July 23, 2021

Contents

S1 International tax treaty system **3**
S1.1 Tax Treaty Rates 3
S1.2 Domestic Tax Rates 3
S1.3 Thin Capitalisation 3

S2 Data sources **3**

S3 List of tax haven countries **4**

S4 List of tax-free countries with zero domestic tax rates **4**

S5 Tax-routing centrality of each layer **5**

S6 Limiting number of changes in income layers **5**

List of Tables

S1 Types of data and sources 6
S2 List of tax haven countries 7
S3 List of tax-free countries in dividends layer 9
S4 List of tax-free countries in royalties layer 10
S5 List of tax-free countries in interest layer 11
S6 Tax-routing centrality of each layer 12

List of Figures

S1 Comparison between direct and shopping routes for restricted transition between layers 12

S1 International tax treaty system

S1.1 Tax Treaty Rates

Tax treaty is basically a bilateral treaty between two countries. If two countries agree on a lower tax rate in the tax treaty than that in domestic tax laws, both countries will withhold tax at the lower tax rate.

S1.2 Domestic Tax Rates

Each country has domestic tax laws for withholding. If a tax treaty is not signed between the two countries, withholding tax is collected according to the domestic tax laws of the source country.

S1.3 Thin Capitalisation

The definition and concept of the thin capitalisatoin can be found at [1] as

> Thin capitalisation refers to the situation in which a company is financed through a relatively high level of debt compared to equity. Tax administrations often introduce rules that place a limit on the amount of interest that can be deducted in calculating the measure of a company's profit for tax purposes. Such rules are designed to counter cross-border shifting of profit through excessive debt, and thus aim to protect a country's tax base.

In the case of interest, tax avoidance can be achieved using interest deduction system (thin capitalisation), but it was not included in our model. We attached the list of each countries' interest deduction data in supporting information (dataset).

S2 Data sources

The tax data used in this paper is a compilation of data on tax treaty rates and domestic tax rates in each country in reports published by PwC, Deloitte, KPMG, and EY in the 2015-2016 fiscal year. The detailed sources are as follows:

- PwC: Worldwide Tax Summaries - Corporate Taxes 2015/16;
- Deloitte: Deloitte International Tax Source (DITS) - Corporate Tax Rates 2016 and Withholding Tax Rates 2016;

- KPMG: Corporate Tax Rates Tables;
- EY: 2016 Worldwide Corporate Tax Guide; The outlook for global tax policy in 2016

We referred to the tax data and notes of each country's tax rates listed in PwC's Worldwide Tax Summaries. The lowest tax rate among withholding tax rates on dividends, interest, and royalties from general commercial transactions, excluding transactions between financial institutions, was listed. Because we assumed the treaty shopping, the lowest tax rates were summarized in the Excel spreadsheets. In the case of royalty data, the lowest tax rate was used among patents, copyrights, etc. Those marked as countries groups (EU, CARICOM, ANDEAN Community, CEMAC) in the PwC data were separated into individual countries and recorded as each country's tax rate.

In case there are difference between PwC data and Deloitte data, we checked and corrected on EY, KPMG, or OECD statistical data, UNCTAD statistical data, and the website of those countries' Ministry of Finance.

The types of data analyzed are the withholding interest tax rate, withholding dividends tax rate, withholding royalties tax rate, a list of countries (regions) joining tax exemption treaties in each countrys tax treaty. We attached the thin capitalisation data in the dataset. The Microsoft's Excel spreadsheet were constructed by the array of 217 countries (regions) by income source. The total number of countries (regions) is 217. Accordingly, dividend income tax rate, interest income tax rate, royalty income tax rate, and domestic withholding tax rates (dividends, interest, and royalties in 217 individual countries), corporate income tax rate, local income tax rate, and thin capitalisation ranges are summarized in (Table S1).

S3 List of tax haven countries

Tax haven countries have been designated by a number of organizations and countries. Here, we present one example of the list of tax haven countries by the Royal Decree of Belgium [2] (Table S2).

S4 List of tax-free countries with zero domestic tax rates

In main manuscript, we identify the countries with zero domestic tax rates, and define those countries as "tax-free countries". Here we present the list of

the countries according to each income type (dividends, royalties, interest) (Table S3-5).

S5 Tax-routing centrality of each layer

In main manuscript, we define and observe the tax-routing centrality (TRC) for identifying central layer or country in treaty-shopping navigation. Here we present the overall TRC value of multiplex network and each single layer respectively (Table S6).

S6 Limiting number of changes in income layers

In main analysis, we consider the treaty-shopping navigation on multiplex networks with three income types of layers. However, it has been not clear whether the multiple-income flows in treaty shopping is generally available due to the lack of fruitful empirical cases. In reality, there would some hurdles to change income types when navigating tax minimizing routes. Therefore, it would be reasonable to introduce some constraints in the algorithm by limiting the number of changes like as considering transition cost. Therefore, we perform the additional calculation of applying a new model with restricted number (one-time) of transition between layers with three cases: (1) dividends to interest (2) royalties to dividends and (3) interest to royalties (Fig.R2).

	Type of Data	Sources
1	(Tax treaty) Dividend income tax rates	PwC, Deloitte, KPMG, EY, OECD, UNCTAD
2	(Tax treaty) Interest income tax rates	PwC, Deloitte, KPMG, EY, OECD, UNCTAD
3	(Tax treaty) Royalty income tax rates	PwC, Deloitte, KPMG, EY, OECD, UNCTAD
4	Corporate income tax rate, local income tax rate in 217 individual countries	Deloitte, PwC, OECD, UNCTAD
5	(Domestic Tax Law) Dividend income tax rates in 217 individual countries	Deloitte, PwC, OECD, UNCTAD
6	(Domestic Tax Law) Interest income tax rates in 217 individual countries	Deloitte, PwC, OECD, UNCTAD
7	(Domestic Tax Law) Royalty income tax rates in 217 individual countries	Deloitte, PwC, OECD, UNCTAD
8	Thin Capitalisation data in 217 individual countries	PwC, Deloitte, KPMG, EY

Table S1: Types of data and sources

no.	country
1	Abu Dhabi
2	Ajman
3	Andorra
4	Anguilla
5	The Bahamas
6	Bahrain
7	Bermuda
8	British Virgin
9	Cayman Islands
10	Dubai
11	Fujairah
12	Guernsey
13	Isle of
14	Jersey
15	Jethou
16	Maldives Islands
17	Micronesia
18	Moldavia
19	Monaco
20	Montenegro
21	Nauru
22	Palau
23	Ras al
24	Saint-Bartholomew
25	Sark
26	Sharjah
27	Turks and
28	Umm al
29	Vanuatu

30	Wallis-and-Futuna

Table S2: Tax Haven List from the Royal Decree of Belgium

no.	country
1	Andorra
2	Anguilla
3	Aruba
4	Bahamas
5	Bahrain
6	Barbados
7	Bermuda
8	Bosnia-Herzegovina
9	Brazil
10	British Virgin Islands
11	Brunei
12	Cayman Islands
13	Curaao
14	Cyprus
15	Denmark
16	Ecuador
17	Estonia
18	Gibraltar
19	Guernsey
20	Hong Kong SAR
21	Hungary
22	Iran
23	Iraq
24	Isle of Man
25	Jersey
26	Jordan
27	Kosovo
28	Kuwait
29	Latvia
30	Libya
31	Liechtenstein
32	Lithuania
33	Luxembourg
34	Macao SAR
35	Madagascar
36	Malaysia
37	Maldives

38	Malta
39	Mauritius
40	Monaco
41	Myanmar
42	Oman
43	Palau
44	Qatar
45	Saint Lucia
46	Saint Vincent & the Grenadines
47	Singapore
48	Sint Maarten
49	Slovakia
50	Sudan
51	Sweden
52	Syria
53	Turks & Caicos
54	United Arab Emirates
55	United Kingdom
56	Vanuatu
57	Vietnam

Table S3: List of tax-free countries in dividends layer

no.	country
1	Anguilla
2	Aruba
3	Bahamas
4	Bahrain
5	Bermuda
6	British Virgin Islands
7	Burkina Faso
8	Cayman Islands
9	Curaao
10	Gibraltar
11	Guernsey
12	Guinea Bissau
13	Hungary

14	Iraq
15	Isle of Man
16	Jersey
17	Kuwait
18	Latvia
19	Libya
20	Liechtenstein
21	Luxembourg
22	Macao SAR
23	Malta
24	Monaco
25	Netherlands
26	Norway
27	Palau
28	Sint Maarten
29	Sweden
30	Switzerland
31	Turks & Caicos
32	United Arab Emirates
33	Vanuatu

Table S4: List of tax-free countries in royalties layer

no.	country
1	Anguilla
2	Aruba
3	Bahamas
4	Bahrain
5	Bermuda
6	British Virgin Islands
7	Cayman Islands
8	Gibraltar
9	Guernsey
10	Hungary
11	Isle of Man
12	Jersey
13	Kuwait

14	Latvia
15	Libya
16	Liechtenstein
17	Luxembourg
18	Macao SAR
19	Malta
20	Monaco
21	Netherlands
22	Norway
23	Palau
24	Sint Maarten
25	Sweden
26	Switzerland
27	Turks & Caicos
28	United Arab Emirates
29	Vanuatu
30	Hong Kong SAR
31	Andorra
32	Cyprus
33	Estonia
34	Maldives
35	Oman
36	Germany
37	Zimbabwe
38	Austria
39	Finland
40	Denmark
41	Greenland
42	France

Table S5: List of tax-free countries in interest layer

layer	TRC^{direct}	$TRC^{shopping}$	diff
Dividends	0.89	0.94	0.05
Royalties	0.86	0.93	0.07
Interest	0.87	0.93	0.06
Multiplex	0.91	0.96	0.05

Table S6: Tax routing centrality of each layer

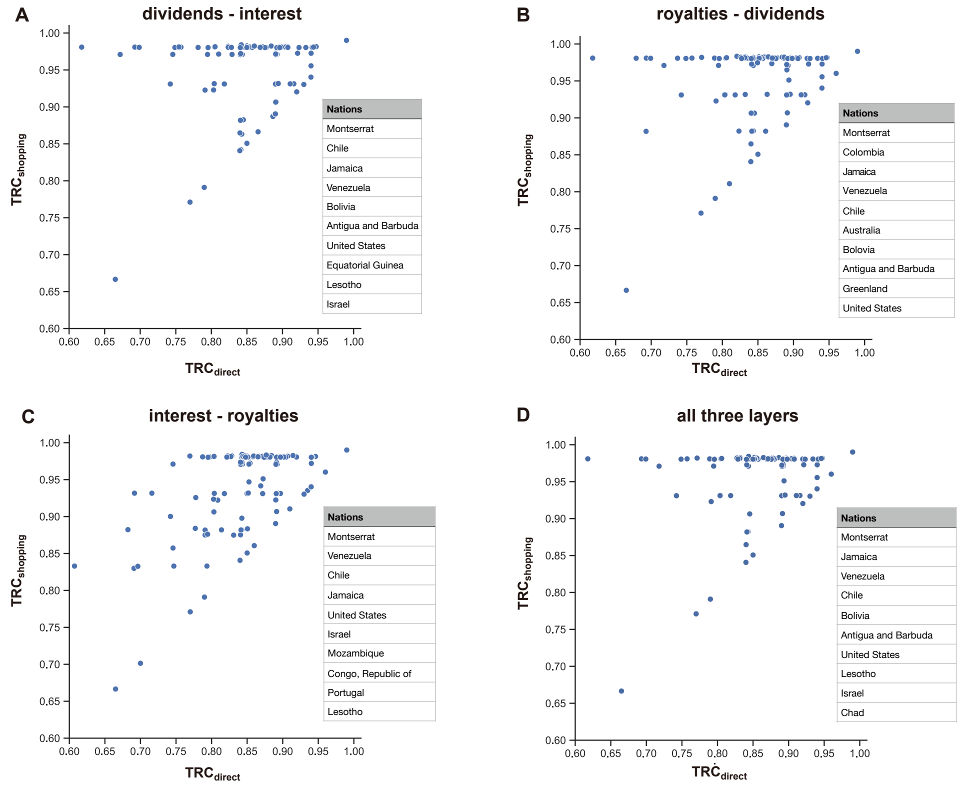

Figure S1: Comparison between direct and shopping routes for restricted transition between (A) dividends to interest, (B) royalties to dividends, (C) interest to royalties, and (D) all transition-allowed (full multiplex) cases.

References

[1] OECD, THIN CAPITALISATION LEGISLATION - A BACKGROUND PAPER FOR COUNTRY TAX ADMINISTRATIONS, August 2012. pp.3-7.

[2] Wendy Zeldin, Belgium: List of Tax Havens Gazetted, Global Legal Monitor, US Library of Congress. May 21, 2010.
