# Supplementary figures and images for: Navigating optimal treaty-shopping routes using a multiplex network model
Source: PLoS One. 2021 Aug 27;16(8):e0256764. doi: 10.1371/journal.pone.0256764 (PMC8396775; doi:10.1371/journal.pone.0256764)

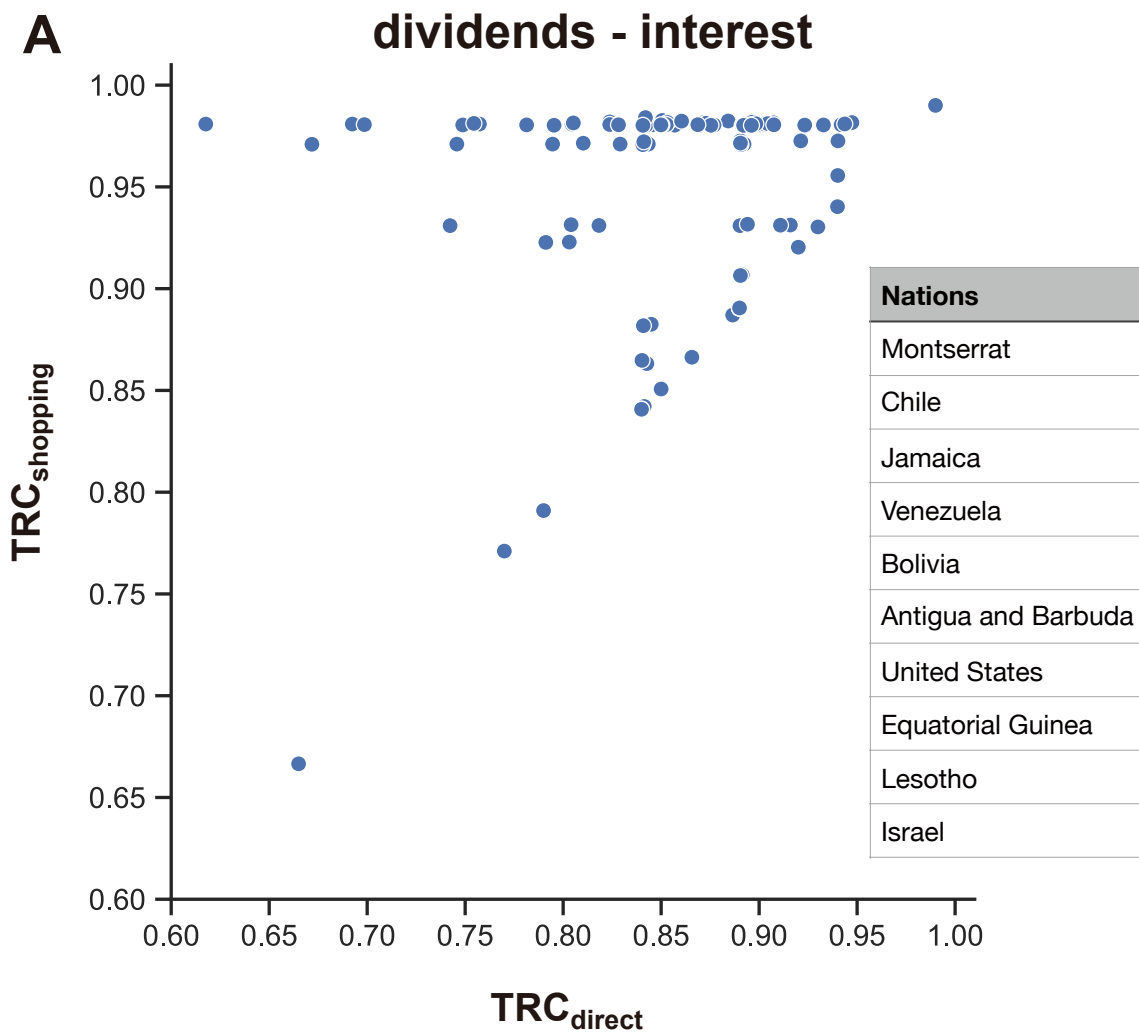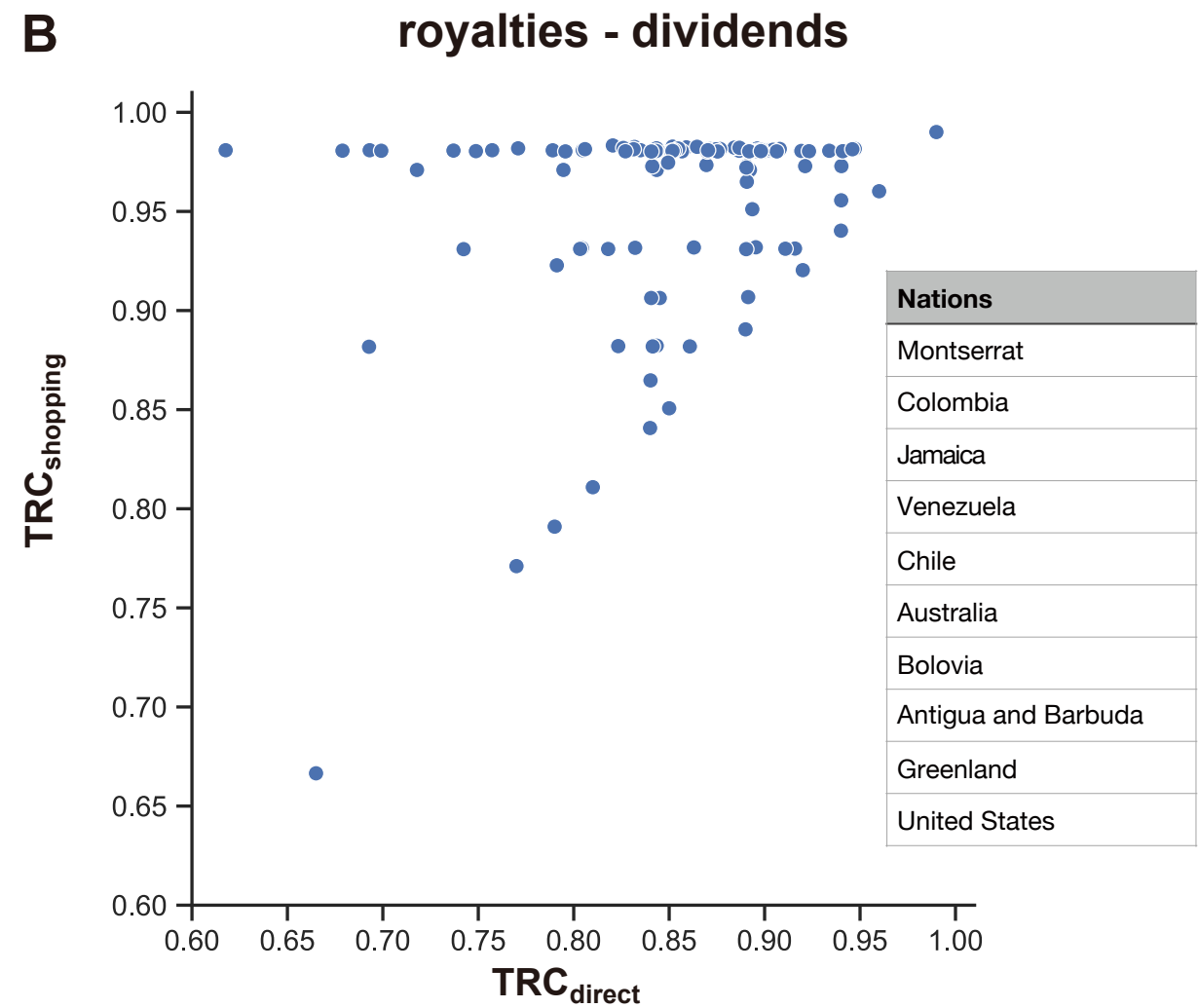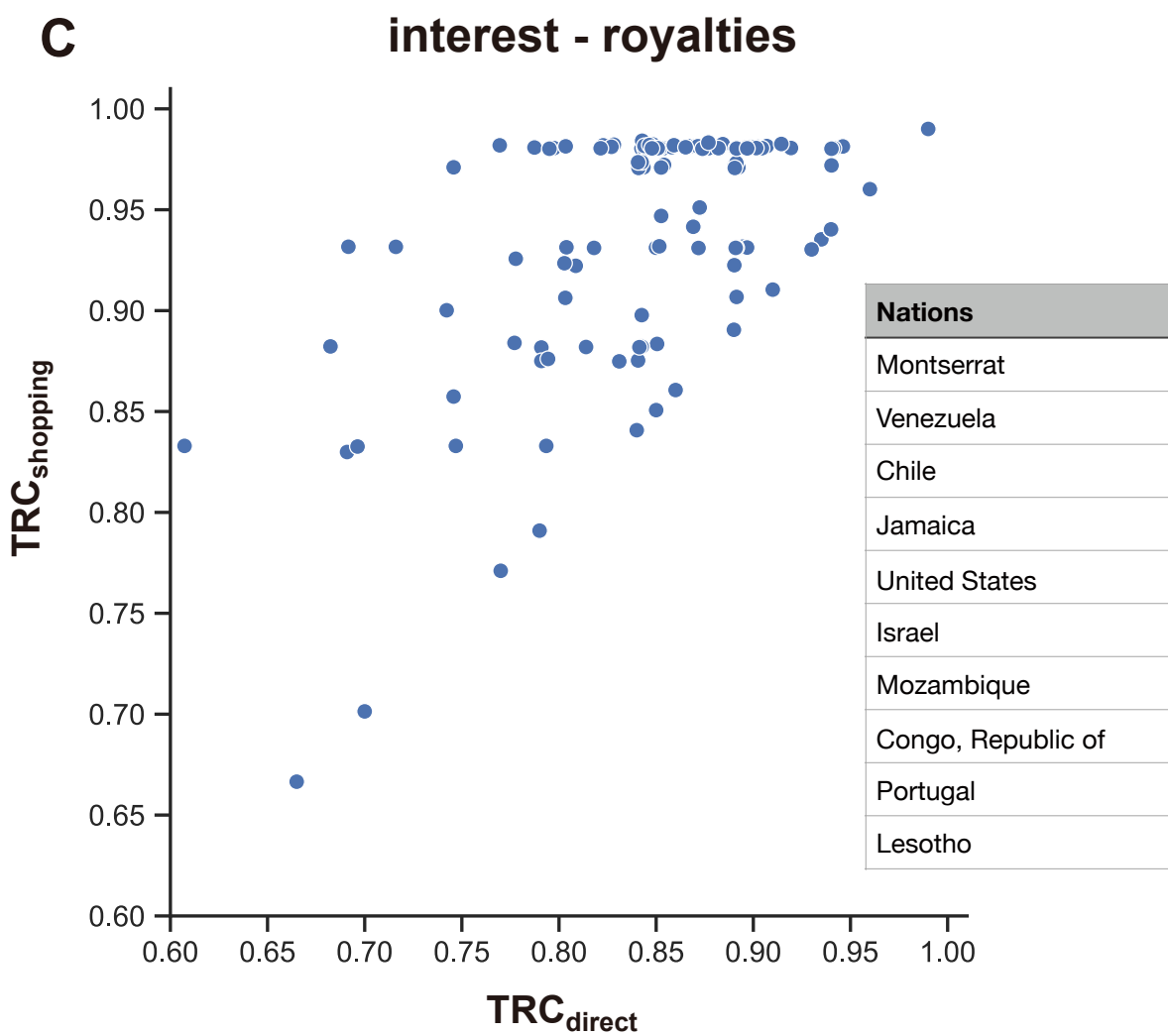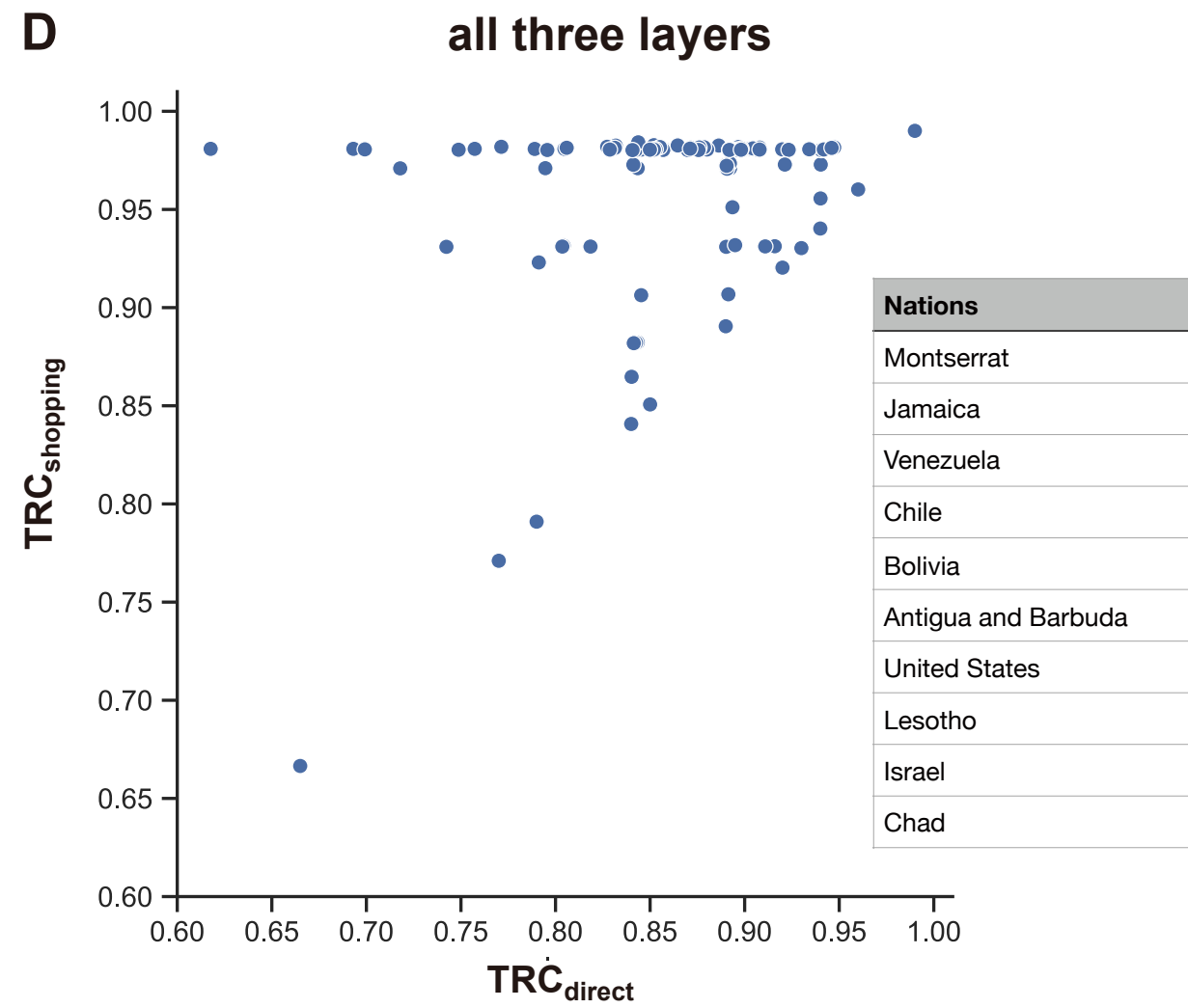

Supplement: S1 Fig — (PDF) [file pone.0256764.s002.pdf]
